# Assessment of Outcomes of Inpatient or Clinic-Based vs Home-Based Rehabilitation After Total Knee Arthroplasty: A Systematic Review and Meta-analysis

**DOI:** 10.1001/jamanetworkopen.2019.2810

**Published:** 2019-04-26

**Authors:** Mark A. Buhagiar, Justine M. Naylor, Ian A. Harris, Wei Xuan, Sam Adie, Adriane Lewin

**Affiliations:** 1 Catholic Diocese of Parramatta, Parramatta, Australia; 2 South West Sydney Clinical School, University of New South Wales, Sydney, Australia; 3 Whitlam Orthopaedic Research Centre, Liverpool, Australia; 4 South Western Sydney Local Health District, Sydney, Australia; 5 Ingham Institute of Applied Medical Research, Liverpool, Australia

## Abstract

**Importance:**

Recent publication of the largest trials to date investigating rehabilitation after total knee arthroplasty (TKA) necessitate an updated evidence review.

**Objective:**

To determine whether inpatient or clinic-based rehabilitation is associated with superior function and pain outcomes after TKA compared with any home-based program.

**Data Sources:**

MEDLINE, Embase, CINAHL, and PubMed were searched from inception to November 5, 2018. Search terms included *knee arthroplasty*, *randomized controlled trial*, *physiotherapy*, and *rehabilitation*.

**Study Selection:**

Published randomized clinical trials of adults who underwent primary unilateral TKA and commenced rehabilitation within 6 postoperative weeks in which those receiving postacute inpatient or clinic-based rehabilitation were compared with those receiving a home-based program.

**Data Extraction and Synthesis:**

Two reviewers extracted data independently and assessed data quality and validity according to the PRISMA guidelines. Data were pooled using a random-effects model. Data were analyzed from June 1, 2015, through June 4, 2018.

**Main Outcomes and Measures:**

Primary outcomes were mobility (6-minute walk test [6MWT]) and patient-reported pain and function (Oxford knee score [OKS] or Western Ontario and McMaster Universities Osteoarthritis Index) reported at 10 to 12 postoperative weeks. The GRADE assessment (Grading of Recommendations, Assessment, Development, and Evaluation) was applied to the primary outcomes.

**Results:**

Five unique studies involving 752 unique participants (451 [60%] female; mean [SD] age, 68.3 [8.5] years) compared clinic- and home-based rehabilitation, and 1 study involving 165 participants (112 [68%] female; mean [SD] age, 66.9 [8.0] years) compared inpatient and home-based rehabilitation. Low-quality evidence showed no clinically important difference between clinic- and home-based programs for mobility at 10 weeks (6MWT favoring home program; mean difference [MD], −11.89 m [95% CI, −35.94 to 12.16 m]) and 52 weeks (6MWT favoring home program; MD, −25.37 m [95% CI, −47.41 to −3.32 m]). Moderate-quality evidence showed no clinically important difference between clinic- and home-based programs for patient-reported pain and function at 10 weeks (OKS MD, −0.15 [95% CI, −0.35 to 0.05]) and 52 weeks (OKS MD, 0.10 [95% CI, −0.14 to 0.34]).

**Conclusions and Relevance:**

Based on low- to moderate-quality evidence, no superiority of clinic-based or inpatient programs compared with home-based programs was found in the early subacute period after TKA. This evidence suggests that home-based rehabilitation is an appropriate first line of therapy after uncomplicated TKA for patients with adequate social supports.

## Key Points

**Question:**

Is inpatient or clinic-based rehabilitation associated with superior outcomes after total knee arthroplasty compared with home programs?

**Findings:**

This systematic review and meta-analysis included 5 unique studies involving 752 unique participants comparing clinic- and home-based rehabilitation and 1 study comparing inpatient rehabilitation with a home-based program. Based on low- to moderate-quality evidence, no associations between settings, no clinically important differences for mobility or patient-reported pain and function at 10 and 52 postoperative weeks, and no significant differences in quality of life or range of motion were found.

**Meaning:**

For adults who underwent total knee arthroplasty, clinic or inpatient vs home-based rehabilitation appeared to offer no clinically important advantages.

## Teaser

This systematic review and meta-analysis assesses whether inpatient or clinic-based rehabilitation is associated with superior function and pain outcomes compared with any home-based rehabilitation among adult patients who underwent total knee arthroplasty.

## Introduction

Total knee arthroplasty (TKA) was the most frequently performed inpatient operating room procedure in the United States in 2012.^1^ From 2003 to 2012, the incidence of TKA increased from 145.4 to 223.0 per 100 000 population (a 4.9% mean annual increase), with the total number performed in the United States projected to increase from 711 000 in 2011 to 3.48 million by 2030.^2^ Similarly in Australia, the incidence increased from 108.3 per 100 000 population in 2003 to 222.3 per 100 000 population in 2017, with more than 54 000 TKAs performed in 2017.^3^

The increased volume of surgery constitutes a significant burden on the acute health care budget, but because the surgery is typically followed by a protracted rehabilitation period, the latter can add significantly to the cost of care. Several studies^4,5,6^ describe a significant cost differential among rehabilitation pathways involving inpatient rehabilitation after TKA, ranging from a 5-fold to a 26-fold cost differential between a rehabilitation pathway that included inpatient therapy and one that did not despite no differences in outcomes between groups. Concern about the total episode-of-care costs for arthroplasty, including the rehabilitation period, has led to the introduction of bundled payments in the United States, consisting of a single bundled payment to health care organizations for all services related to the TKA to 90 days after surgery.^7,8^ This payment approach has subsequently driven health care providers to reconsider the use of the more expensive inpatient rehabilitation pathways.^7^

Outside inpatient rehabilitation, the setting, cost, and modes of provision vary greatly when rehabilitation is delivered in the community.^6,9^ Available options include one-to-one or group-based interventions (land or water) and various iterations of home-based care, including domiciliary programs (physiotherapy visits in the home), telerehabilitation, or more simple monitored (via occasional clinic visits or telephone contact) or unmonitored home programs.^9,10,11,12,13^ Previous systematic reviews of randomized clinical trials^14,15^ have concluded that no single setting—clinic- or home-based, in water or on land—appears to be associated with better recovery across a range of outcomes. Despite this finding, to date, no evidence-based clinical practice guideline exists to promote the use of home-based programs after uncomplicated TKA. Trials published since the aforementioned reviews,^16,17,18^ however, have included new comparisons (inpatient and 3-arm trials) and constitute the largest TKA rehabilitation trials to date. Thus, a more contemporary review is warranted, potentially as a precursor to development of a much-needed clinical practice guideline.

The aim of this systematic review and meta-analysis was to investigate the importance of the rehabilitation setting on outcomes for adults after elective, primary, unilateral TKA. Specifically, we aimed to determine whether inpatient or clinic-based rehabilitation is associated with superior function and pain outcomes after TKA compared with any home-based physiotherapy program (monitored or unmonitored, or domiciliary [physiotherapy home visitation]). Superiority was defined as a change considered to be clinically important for each outcome assessed.

## Methods

This systematic review of randomized clinical trials follows the methods described in the *Cochrane Handbook for Systematic Reviews of Interventions, Version 5.1.0*^19^ and is reported in accordance with the Preferred Reporting Items for Systematic Reviews and Meta-analyses (PRISMA) reporting guideline.^20^ The protocol was updated to include the GRADE (Grading of Recommendations, Assessment, Development and Evaluation) component for assessing quality of evidence.^21^

### Eligibility Criteria

#### Types of Studies

Published randomized clinical trials were eligible for inclusion. We excluded studies reported only as abstracts if adequate data could not be obtained from the authors, studies in which TKA data could not be separated from other procedures (eg, total hip arthroplasty), and studies for which we were unable to obtain potentially relevant data from the authors on request. No language restrictions were applied.

#### Types of Participants

We included studies of adults (age ≥18 years) who had undergone a primary unilateral TKA and commenced rehabilitation within 3 months of surgery. We excluded studies of unicompartmental surgery, revision TKA, or TKA secondary to trauma.

#### Types of Interventions

We included studies investigating rehabilitation after TKA in which patients who had received postacute inpatient or clinic-based rehabilitation were compared with others who had received a monitored or an unmonitored home-based or domiciliary program after discharge from the acute-care facility. We excluded telerehabilitation because other reviews in progress during conduct of our study and subsequently published^22,23,24^ have investigated this option.

### Outcomes

The goal of physiotherapy-based rehabilitation after TKA is to improve physical function, including walking, activities of daily living, and knee mobility. We grouped outcomes into the following categories that broadly reflect these goals: physical performance test results (6-minute walk test [6MWT],^25^ measured as laps walked on a flat surface in 6 minutes; walking speed, stair ascent and descent tests, and chair rise test), patient-reported pain and function (Oxford knee score^26^ [OKS; range, 0-48, with higher scores indicating best outcomes], Knee Injury and Osteoarthritis Outcome Score^27^ [range, 0-100, with higher scores indicating worse outcomes], and Western Ontario and McMaster Universities Osteoarthritis Index^28^ [5 items for pain, 2 for stiffness, and 17 for functional limitation, with higher scores indicating worse outcomes]), generic health-related quality-of-life measures (12- and 36-Item Short Form Health Surveys and EuroQol-5D^29^), and knee range of motion (ROM), expressed as active or passive ROM, extension, and/or flexion.

### Primary and Secondary Outcomes

Primary outcomes were mobility (6MWT) and patient-reported pain and function (OKS) measured at 10 to 12 postoperative weeks. Secondary outcomes included mobility and patient-reported pain and function, knee ROM, postoperative complications, and health-related quality of life measured at 10, 26, and/or 52 weeks.

### Identification and Selection of Studies

We searched Embase, PubMED, MEDLINE, and CINAHL from inception to June 19, 2018, using search terms that included *knee arthroplasty*, *randomized controlled trial*, *physiotherapy* and related terms, and *rehabilitation* (eMethods 1 in the Supplement). We later scanned references of all included studies.

Two reviewers (M.A.B. and J.M.N.) independently screened titles and abstracts of the search output to identify studies suitable for further scrutiny. They discussed inconsistencies in the screening process before a decision was made to review the full text. The same reviewers then screened full-text articles to determine inclusion in the review. Discrepancies in the final list were discussed, and a consensus was reached for all articles.

### Assessment of Study Quality, Risk of Bias, and GRADE Assessment

Two reviewers (M.A.B. and J.M.N.) independently assessed study quality using the Cochrane Collaboration Risk of Bias tool,^30^ which includes the following variables: random allocation sequence, allocation concealment, blinding (of patients, therapists, and outcome assessors), attrition (loss to follow-up and intention-to-treat analysis), and selective outcome reporting. Disagreements in risk of bias were resolved by discussion, or, when necessary, a third person arbitrated. Included studies were also assessed using the Physiotherapy Evidence Database scale,^31^ used to identify trials that are more likely to be valid and to contain sufficient information to guide clinical practice.

Two reviewers (S.A. and A.L.) independently used the GRADE component to categorize the quality and strength of the evidence as high, moderate, low, and very low for the 6MWT and patient-reported pain and function at 10 to 12 postoperative weeks (the primary outcomes) and at 52 weeks.^32^ Disagreements were resolved by consensus between the 2 reviewers. To ensure reproducibility and consistency, the reviewers used a checklist to rate each component of the GRADE assessment.^33^ We used GRADEpro software to create summary of findings tables.^34^ Because 4 of the investigators (M.A.B., J.M.N., I.A.H., and W.X.) were involved in randomized clinical trials relevant to this review, the GRADE assessment was undertaken by reviewers not involved in any of the included studies.

### Data Extraction

Four reviewers (M.A.B., J.M.N., S.A., and A.L.) independently extracted data. We collected data related to participants (diagnosis, age, sex, and body mass index); country; study dates; inclusion and exclusion criteria; setting, timing, duration, and intensity of the intervention and comparison (control) conditions; duration of follow-up; losses to follow-up and reasons; and outcomes. Means (SDs) were extracted for outcomes reported as continuous variables. Proportions were extracted for categorical outcomes. Appropriate conversions were applied when outcomes were reported as medians and interquartile ranges or means and 95% CIs.^19^

For studies with incomplete data, we attempted to contact the corresponding author. We also asked whether any outcomes not reported in their publications had been collected. When authors of included studies were unable to provide additional data, all available data were included in the review. If data had been provided by authors to other reviewers in published reviews, these were included in the analyses in the case of failure to retrieve data from the primary source and acknowledged appropriately. Authors of included studies were also contacted when there was incomplete reporting of data. Where possible, we used data from intention-to-treat analyses in our calculations to determine between-group differences.

For studies with 3 randomized arms, we adopted a strategy described by Higgins and Green^19^ of including each comparison separately but with the shared intervention group divided evenly among the comparisons. For continuous outcomes, only the total number of participants was divided, with the means (SDs) left unchanged.

### Statistical Analysis

#### Measures of Treatment Effect

Data were analyzed from June 1, 2015, through June 4, 2018. We used the mean differences (MDs) and 95% CIs for continuous outcomes with the same units (eg, 6MWT). We presented continuous outcomes with different units as standardized MDs and 95% CIs. Categorical outcomes were expressed as a number with percentage.

#### Data Synthesis

The 2 main comparisons (clinic- vs home-based and inpatient vs home-based) were considered separately. Where possible, we pooled data using random-effects meta-analysis.^35^ Because the standardized MD can be artifactually affected by correlation between baseline and follow-up measurements when including the SD of change along with the SD of absolute values, we used the baseline SD for change score values when combining change scores and absolute values. For dichotomous outcomes, we used a pooled odds ratio.

We used the *I*^2^ statistic to assess statistical heterogeneity among included studies. We planned to explore publication bias using funnel plots if we had a minimum of 10 included studies, but the number found did not reach this. We used RevMan software (version 5.3)^36^ to compile data and perform statistical analyses.

## Results

### Results of the Search

**Figure 1.**
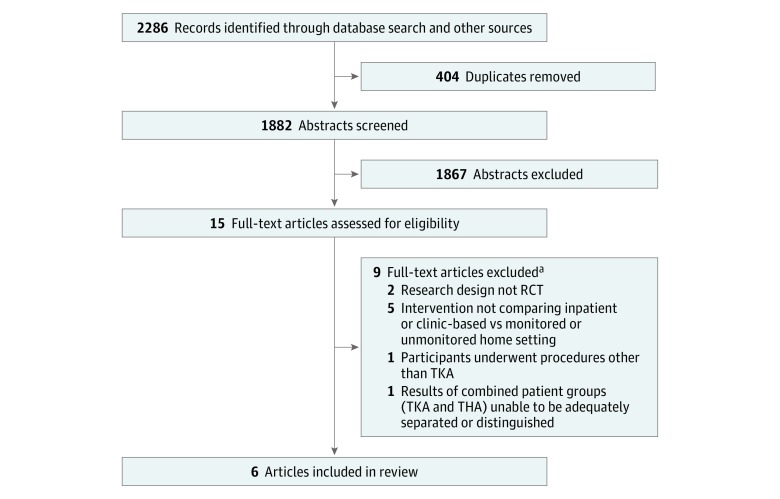
Flow of Studies Through the Review RCT indicates randomized clinical trial; THA, total hip arthroplalsty; and TKA, total knee arthroplasty. ^a^Studies may have been excluded for failing to meet more than 1 inclusion criterion.

The search strategy yielded 2286 references. After duplicates were removed using the duplicate removal program within EndNote commercial reference management software (Clarivate Analytics) and titles and abstracts were screened, we retrieved 15 studies for evaluation, of which 9 studies were excluded (eMethods 2 in the Supplement). Six eligible randomized clinical trials were included in the review (Table 1),^16,17,37,38,39,40^ 5 of which were included in the meta-analysis (Figure 1).^17,37,38,39,40^

**Table 1.** Summary of Included Studies

Source (Country)	No. of Participants	Diagnosis	Primary Focus of Intervention	Setting	Intervention Condition	Control Condition	Outcome Assessment	Follow-up Point, wk
Buhagiar et al,^16^ 2017 (Australia)	165	TKA and osteoarthritis	Simple and advanced functional, aerobic, and strengthening exercises	Inpatient rehabilitation and home-based groups	Inpatient rehabilitation and home: 10 d of twice-daily inpatient PT; 2-3 OP physiotherapy sessions for 10 wk, starting 2-3 wk after surgery	Home: 2-3 OP PT sessions for 10 wk, starting 2-3 wk after surgery	6MWT, 10MWT, OKS, knee ROM ≥100°, KOOS, EuroQol-5D, PO complications	10, 26, and 52
Ko et al,^17^ 2013 (Australia)	249	TKA and osteoarthritis	Simple and advanced functional, aerobic, and strengthening exercises	1:1:1 Randomized clinic-, group clinic–, and home-based groups	Clinic and group clinic: 2 OP PT sessions per week for 6 wk, starting 2-3 wk after surgery	Home: 2 OP PT visits with follow-up telephone call for 6 wk, starting 2-3 wk after surgery	OKS, WOMAC function, knee ROM, 6MWT, timed stairs ascent and descent, SF-12 physical and mental scores, PO complications	2, 10, 26, and 52
Kramer et al,^37^ 2003 (Canada)	160	TKA and osteoarthritis	Simple and advanced strengthening and ROM exercises	Clinic- and home-based groups	Clinic: two 1-h OP PT sessions per week for 10 wk, starting 1 wk after surgery. Home: exercise program upgraded by treating therapist	Home: monitored via 2 telephone calls between 2-12 wk after surgery	WOMAC, SF-36, Knee Society Scale, timed stair ascent and descent, 6MWT	12, 26, and 52
Madsen et al,^38^ 2013 (Denmark)	80	TKA and osteoarthritis	Strengthening, endurance, functional, and ROM exercises	Group clinic- and home-based groups	Group clinic: 2 PT sessions per week for 6 wk, starting 4-8 wk after surgery. Strength and endurance training and patient education and discussion. Home: exercises twice weekly with strength training, endurance training on exercise bike, walking, and balance training	Home: 2 OP PT visits in total, with additional OP visits allowed (not exceeding 12) for participants with physical limitations	OKS, SF-36 physical function, EuroQol-5D, knee ROM, peak leg extensor power, balance test, 10MWT, sit-to-stand tests, VAS pain during leg extensor power test	12 and 26
Mockford et al,^39^ 2008 (Northern Ireland)	143	TKA and osteoarthritis or rheumatoid arthritis	Functional, strengthening, and ROM exercises	Clinic- and home-based groups	Clinic: home exercise regime to follow on discharge; PT sessions for 6 wk, starting within 3 wk of hospital discharge	Given home exercise regime to follow on discharge, with no OP PT	OKS, Bartlett Patellar Score, SF-12, PO complications	12 and 52
Rajan et al,^40^ 2004 (England)	120	TKA and monoarticular arthrosis	No information on primary focus of intervention	OP clinic vs unmonitored home-based group	Clinic: PT sessions (mean, 4-6) after discharge from hospital. No information on program content.	Home: No information on program. Patients from both groups given a home exercise regime on discharge	ROM	12, 26, and 52

Abbreviations: KOOS, Knee Injury and Osteoarthritis Outcome Score; OKS, Oxford knee score; OP, outpatient; PO, postoperative; PT, physiotherapy; ROM, range of motion; SF-12, 12-Item Short Form Health Survey; SF-36, 36-Item Short Form Health Survey; TKA, total knee arthroplasty; VAS, visual analog scale; WOMAC, Western Ontario and McMaster Universities Osteoarthritis Index; 6MWT, 6-minute walk test; 10MWT, 10-minute walk test.

### Included Studies

Five unique studies^17,37,38,39,40^ with a total of 752 unique participants (451 [60%] female; mean [SD] age, 68.3 [8.5] years) included in the meta-analysis compared outpatient rehabilitation (individual and/or group) with home-based rehabilitation (monitored or unmonitored). The sixth study^16^ with 165 patients (112 [68%] female; mean [SD] age, 66.9 [8.0] years) compared inpatient rehabilitation with home-based rehabilitation monitored by a health care professional. This study was included in a qualitative synthesis only. In all studies, rehabilitation commenced within 3 months of surgery, and participants were followed up for 26 to 52 weeks. Among studies that reported diagnostic data, the most common diagnosis was osteoarthritis. The mean (SD) age of study patients ranged from 66.2 (8.2) to 70.9 (SD not provided) years among studies reporting age. All studies excluded patients with complications in the acute postoperative period. Four studies reported patient adherence to the program (88%^16^; 77%^17^; 96%^37^; and 61%^38^) (Table 1). One study^17^ included in this meta-analysis had 3 randomized arms. We included each comparison separately (in this case, outpatient group–based vs home-based rehabilitation and outpatient one-to-one therapy vs home-based rehabilitation) but with the shared intervention group (ie, the home-based treatment arm) divided evenly among the comparisons as previously described in the data extraction section.^19^

### Risk of Bias in Included Studies

**Figure 2.**
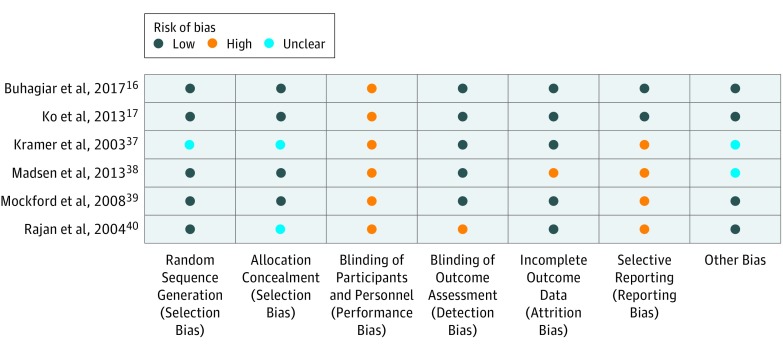
Cochrane Risk of Bias Table Study quality was assessed using the Cochrane Collaboration Risk of Bias Tool.^30^

As shown in Figure 2, 5 studies^16,17,38,39,40^ used adequate methods for generating the randomization sequence, with the sixth study^37^ not providing this information. Four studies^16,17,38,39^ described the use of adequate methods to conceal allocation. Blinding of participants and therapists was not possible in any of the studies owing to the nature of the intervention, but all included studies blinded assessors of objective outcomes to group allocation. Two studies^16,17^ were free of selective outcome reporting. Risk of bias was present in 2 studies^37,38^ owing to uneven losses to follow up. Physiotherapy Evidence Database scores assessing study quality ranged from 5 to 8 (maximum of 10) (eTable 1 in the Supplement). We did not use funnel plots to explore publication bias owing to the small number of included studies.

### Intervention Outcomes and Comparisons

The results for all outcomes and comparisons of clinic-based rehabilitation with monitored or unmonitored home-based programs are summarized in eTable 2 in the Supplement. Because only 1 study assessed inpatient rehabilitation,^16^ meta-analysis was not possible, and a brief narrative summary is provided.

### Primary Outcomes

#### Mobility

Two studies^17,37^ reported the 6MWT at 1 or more follow-up points (eTable 2 and eFigure 1 in the Supplement). Participants who received clinic-based rehabilitation (n = 231) had walked an MD in 6MWT of −11.89 m (95% CI, −35.94 to 12.16 m; *P* = .33) compared with those who received a home-based program (n = 142) at 10 to 12 weeks and an MD in 6MWT of −3.05 m (95% CI, −29.75 to 23.66 m; *P* = .82) compared with those receiving a home-based program in both studies with 243 participants at 26 weeks. At 52 weeks, participants who had undergone clinic-based rehabilitation had walked an MD in 6MWT of −25.37 m (95% CI, −47.41 to −3.32 m; *P* = .02) compared with participants who had undergone clinic-based rehabilitation. Based on GRADE assessment, low-quality evidence suggests that there may be no clinically important difference between clinic- and home-based programs for mobility at 10 and 52 weeks (Table 2). Minimal heterogeneity was found across studies reporting mobility outcomes.

**Table 2.** GRADE Component for Clinic-Based Compared With Home-Based Rehabilitation for Total Knee Arthroplasty at 10 and 52 Weeks

Outcomes	No. of Participants (No. of RCTs) in Follow-up	Certainty of the Evidence, GRADE Component^a^	Anticipated Absolute Effects^b^	
			Mean Value With Home-Based Rehabilitation	MD With Clinic-Based Rehabilitation (95% CI)
At 10- to 12-wk follow-up				
Mobility assessed with 6MWT	373 (3)	Low^c^^,^^d^	371.0 m	−11.89 m (−35.94 to 12.16 m)
Pain and function assessed with OKS^e^	457 (4)	Moderate^c^^,^^f^	NE	−0.15 points (−0.35 to 0.05 points)^g^
At 52-wk follow-up				
Mobility assessed with 6MWT	369 (3)	Low^c^^,^^d^	414.6 m	−25.37 m (−47.41 to −3.32 m)
Pain and function assessed with OKS^e^	388 (3)	Moderate^c^^,^^f^	NE	0.10 points (−0.14 to 0.34 points)^g^

Abbreviations: GRADE, Grading of Recommendations, Assessment, Development and Evaluation; MD, mean difference; NE, not estimable; OKS, Oxford knee score; RCT, randomized clinical trial; 6MWT, 6-minute walk test.

^a^ High indicates very confident that the true effect lies close to that of the estimate of the effect; moderate, moderately confident that the true effect is likely to be close to the estimate of the effect, but with a possibility that it is substantially different; low, limited confidence and the true effect may be substantially different from the estimate of the effect; and very low, very little confidence and the true effect is likely to be substantially different from estimate of effect.

^b^ The risk in the intervention group (and its 95% CI) is based on the assumed risk in the comparison group and the relative effect of the intervention (and its 95% CI).

^c^ Differential loss to follow-up in 1 study: 19% (clinic-based group) and 28% (home-based group).

^d^ Total sample size was less than 400 (optimal information size for continuous outcomes).

^e^ Range of possible scores was 0 to 48, with higher scores indicating best outcomes.

^f^ Outcome assessment was not described in 1 study.

^g^ Standardized MD.

#### Patient-Reported Pain and Function

Three studies^17,38,39^ reported a pain and function outcome at 1 or more follow-up points using the OKS (absolute values or change from baseline). Based on the GRADE component, moderate-quality evidence suggests little or no difference between clinic- and home-based programs for patient-reported pain and function in 457 patients at 10 weeks (MD, −0.15; 95% CI, −0.35 to 0.05) and in 388 patients at 52 weeks (MD, 0.10; 95% CI, −0.14 to 0.34) (Table 2 and eFigure 2 in the Supplement).

### Secondary Outcomes

#### Patient-Reported Quality of Life

Two studies^17,38^ reported quality-of-life outcomes at 1 or more follow-up points using the 12- or 36-Item Short Form Health Survey. No superiority of outcomes was found for patients receiving clinic-based rehabilitation compared with those who received a monitored or unmonitored home-based program in 314 participants at 10 to 12 weeks, 313 participants at 26 weeks, and 242 participants at 52 weeks (eFigure 3 in the Supplement). Less than 50% heterogeneity was found across studies reporting quality-of-life outcomes.

#### Active ROM

Active ROM flexion data suitable for meta-analysis were available from 3 studies^37,39,40^ with 386 participants and for active ROM extension from 1 study^39^ with 143 participants. No benefit was seen for these outcomes at any point (eFigure 4 in the Supplement). Greater heterogeneity was found across studies reporting ROM outcomes.

#### Passive ROM

Two studies^17,38^ with 314 participants reported passive ROM data suitable for meta-analysis (eFigure 5 in the Supplement). No superiority of outcomes in passive ROM was found between randomized groups at 10 to 12, 26, or 52 weeks after surgery.

### Inpatient Rehabilitation vs Home-Based Rehabilitation

A single study^16^ compared inpatient with home-based rehabilitation. In an intention-to-treat analysis, the authors reported no difference in the primary outcome of the 6MWT between the 2 randomized groups. A per protocol analysis of the primary outcome yielded similar results. The unadjusted and adjusted group effects were nonsignificant for all secondary outcomes at 10, 26, and 52 weeks (OKS, knee ROM ≥100°, 10MWT, Knee Injury and Osteoarthritis Outcome Score, and EuroQol-5D score). Per protocol analyses yielded the same results across all points. Moderate-quality evidence suggested that inpatient rehabilitation was not associated with superior mobility and patient-reported pain and functional outcomes compared with the outcomes from a monitored home program; however, this result must be interpreted cautiously because it is based on a single study (Table 3).

**Table 3.** GRADE Component for Inpatient Compared With Home-Based Rehabilitation for Total Knee Arthroplasty at 10 and 52 Weeks

Outcomes	No. of Participants (No. of RCTs) in Follow-up	Certainty of the Evidence, GRADE Component^a^	Anticipated Absolute Effects^b^	
			Mean Value With Home-Based Rehabilitation	MD With Inpatient Rehabilitation (95% CI)
At 10- to 12-wk follow-up				
Mobility assessed with 6MWT	158 (1)	Moderate^c^	383.2 m	3.6 m (−23.2 to 30.4 m)
Pain and function assessed with OKS^d^	157 (1)	Moderate^c^	32.1 points	1.21 points (−1.45 to 3.88 points)
At 52-wk follow-up				
Mobility assessed with 6MWT	150 (1)	Moderate^c^	404.8 m	−13.5 m (−40.7 to 13.6 m)
Pain and function assessed with OKS^d^	160 (1)	Moderate^c^	37.0 points	−0.55 points (−3.21 to 2.1 points)

Abbreviations: GRADE, Grading of Recommendations, Assessment, Development and Evaluation; MD, mean difference; OKS, Oxford knee score; RCT, randomized clinical trial; 6MWT, 6-minute walk test.

^a^ High indicates very confident that the true effect lies close to that of the estimate of the effect; moderate, moderately confident that the true effect is likely to be close to the estimate of the effect, but with a possibility that it is substantially different; low, limited confidence and the true effect may be substantially different from the estimate of the effect; and very low, very little confidence and the true effect is likely to be substantially different from estimate of effect.

^b^ The risk in the intervention group (and its 95% CI) is based on the assumed risk in the comparison group and the relative effect of the intervention (and its 95% CI).

^c^ Only 1 study and a small sample size.

^d^ Range of possible scores was 0 to 48, with higher scores indicating best outcomes.

## Discussion

### Summary of Main Findings

This systematic review and meta-analysis found that, based on low- to moderate-quality evidence, clinic-based rehabilitation after TKA was not associated with superior outcomes compared with a home-based program, whether monitored or unmonitored, when considering mobility, pain, function, quality of life, active knee flexion and extension, and passive knee ROM. Similarly, inpatient rehabilitation after TKA does not deliver superior outcomes compared with monitored home-based rehabilitation when considering mobility, pain, function, quality of life, and knee flexion.

Home-based rehabilitation provided greater mobility (approximately 25 m more in the 6MWT) at 52 weeks compared with a clinic-based program. However, research indicates that this difference is not clinically important. Using a triangulation of methods, including patient-perceived anchor-based thresholds and distribution-based thresholds, Naylor and colleagues^41^ proposed that the threshold for minimal or more improvement for the 6MWT after TKA is expected to range from 26 to 55 m. For patients with chronic obstructive pulmonary disease, Rasekaba and colleagues^42^ determined the minimal clinically important distance for the 6MWT is 54 m, with a similar figure (50 m) determined for a population of older adults and those with stroke by Perera et al.^43^

A single study provided evidence that inpatient rehabilitation is not associated with better mobility and patient-reported pain and function outcomes compared with a monitored home-based program among adults undergoing uncomplicated TKA.^16^ This study reported that inpatient rehabilitation was associated with higher levels of patient-reported satisfaction. Understanding the reason for this finding would be useful for informing alternative models of rehabilitation provision.

No studies included in this review considered whether outcomes of post-TKA rehabilitation delivered in the domiciliary setting differed from those in other rehabilitation settings. One study comparing inpatient with domiciliary rehabilitation^44^ combined data from recipients of total hip and knee arthroplasty and concluded that the combined cohort had no difference in pain, functional outcomes, or patient satisfaction between the 2 treatment groups and that inpatient rehabilitation was not cost-effective. We were not able to obtain individual joint data from the authors, so were not able to include these data in our meta-analysis.

We were also unable to include the largest randomized clinical trial conducted to date concerning rehabilitation after TKA (n = 390).^18^ This study compared usual care with a home-based exercise program; however, usual care consisted of any combination of clinic- or inpatient-based programs, and many in the home-based program also accessed clinic-based care. Thus, we were unable to assign their participants to exclusively home-based or facility-based care. Those authors concluded that a home-based exercise program was not inferior to usual care for a range of patient-reported and objectively measured outcomes, including the Western Ontario and McMaster Universities Osteoarthritis Index, walking speed, and knee ROM.

### Comparison With Other Reviews

A systematic review and meta-analysis^14^ published in 2015 examined the effectiveness of physiotherapy exercise after TKA and found no differences for outpatient compared with home-based physiotherapy exercise for physical function or pain outcomes. A short-term benefit that favored home-based physiotherapy exercise for ROM flexion was not clinically important. These findings are consistent with those of our review.

### Quality of the Evidence

The risk of bias in the 5 studies included in the review was variable. The primary source of potential bias was from uneven losses to follow-up in 2 studies.^37,38^ Another potential source of bias, because of the nature of the intervention, was that participants could not be blinded to their treatment.

### Future Considerations

We identified a number of ongoing randomized trials comparing rehabilitation settings after TKA for future consideration. One, identified via a search in ClinicalTrials.gov,^45^ plans to evaluate unsupervised home exercise with and without a web-based recovery platform compared with traditional outpatient physiotherapy after TKA. Another, with a published protocol^46^ and feasibility study,^47^ will compare clinic-based group physiotherapy with usual (home-based) care. However, the method proposed suggests possible crossover between settings in the latter arm because some patients were referred to physiotherapy services on an individual basis at the discretion of the hospital physiotherapist, orthopedic team, or general physician, with no indication of how many such referrals were made. We also updated our search to November 2018, with no new eligible trials identified.

### Strengths and Limitations

This review has specific strengths. We included only studies in which treatment assignments were randomized, enhancing the strength of the conclusions that could be drawn from the findings. Our review was also comprehensive because we included non–English-language articles in the search strategy, although none were found to be suitable for inclusion.

This review also has several limitations. First, the failure to identify all relevant studies is a common source of bias in systematic reviews. We conducted thorough searches of research databases as well as clinical trial registries, including studies in all languages, using reference list searches of included studies and forward citation tracking, and corresponded with authors of included studies. We identified 1 study that was reported only in a conference proceeding^48^ and compared clinic-based rehabilitation with an unmonitored home-based program. However, that study had significant risk of bias, including failure to mask outcome assessors and incomplete outcome data, and data obtained from the author were insufficient for inclusion in the analysis. Despite these efforts, failing to identify unpublished studies may have introduced bias.

Second, the number of studies in this area of research is small, limiting the precision of the findings and influencing the ability to assess for publication bias graphically or statistically. Third, the method used to divide data from the 3-arm study^17^ only partially overcomes unit-of-analysis error because the resulting comparisons remain correlated,^19^ meaning that the narrowness of the 95% CIs may have been overestimated. Also, we did not consider complications occurring in the subacute care period as part of this review because of uncertainty around the standardization of reporting in this area. However, our conclusions apply to patients who did not experience major complications during the acute-care period that would have prohibited their involvement in the programs prescribed.

## Conclusions

Several clinical trials have investigated the influence of setting on the effectiveness of rehabilitation delivered in the early subacute phase after TKA. This review found consistent evidence suggesting no clinically important differences between clinic-based or inpatient rehabilitation after TKA compared with a home-based program across a range of outcomes. Care that aligns with this evidence would incorporate home-based rehabilitation as the first line of therapy, reserving the more intensely supervised approaches for the most impaired patients or those without adequate social supports. In our view, the development of an evidence-based clinical practice guideline appears to be the next step in synthesizing this literature and aligning practice with the most up-to-date evidence.

## Notes

Supplement. **eMethods 1.** Search Strings for Systematic Review and Meta-analysis **eMethods 2.** Studies Excluded After Full-Text Review **eTable 1.** PEDro Scores of Included Studies **eTable 2.** Meta-analysis Data **eFigure 1.** Mobility **eFigure 2.** Patient-Reported Pain and Function **eFigure 3.** Patient-Reported Quality of Life **eFigure 4.** Active Range of Motion **eFigure 5.** Passive Range of Motion Change From Baseline
