# Supplementary material for: Assessment of Outcomes of Inpatient or Clinic-Based vs Home-Based Rehabilitation After Total Knee Arthroplasty: A Systematic Review and Meta-analysis
Source: JAMA Netw Open. 2019 Apr 26;2(4):e192810. doi: 10.1001/jamanetworkopen.2019.2810 (PMC6487570; doi:10.1001/jamanetworkopen.2019.2810)
Supplement: Supplement. — eMethods 1. Search Strings for Systematic Review and Meta-analysis eMethods 2. Studies Excluded After Full-Text Review eTable 1. PEDro Scores of Included Studies eTable 2. Meta-analysis Data eFigure 1. Mobility eFigure 2. Patient-Reported Pain and Function eFigure 3. Patient-Reported Quality of Life eFigure 4. Active Range of Motion eFigure 5. Passive Range of Motion Change From Baseline [file jamanetwopen-2-e192810-s001.pdf]

Supplementary Online Content

Buhagiar MA, Naylor JM, Harris IA, Xuan W, Adie S, Lewin A. Assessment of outcomes of inpatient or clinic-based vs home-based rehabilitation after total knee arthroplasty: a systematic review and meta-analysis. *JAMA Netw Open*. 2019;2(4):e192810. doi:10.1001/jamanetworkopen.2019.2810

eMethods 1. Search Strings for Systematic Review and Meta-analysis
eMethods 2. Studies Excluded After Full-Text Review
eTable 1. PEDro Scores of Included Studies
eTable 2. Meta-analysis Data
eFigure 1. Mobility
eFigure 2. Patient-Reported Pain and Function
eFigure 3. Patient-Reported Quality of Life
eFigure 4. Active Range of Motion
eFigure 5. Passive Range of Motion Change From Baseline

This supplementary material has been provided by the authors to give readers additional information about their work**.**

© 2019 Buhagiar MA et al. *JAMA Network Open.*

eMethods 1. Search Strings for Systematic Review and Meta-analysis

Medline

No	Searches
1	exp Rehabilitation/
2	(rehabilit* or habilitat*).mp.
3	exp Physical Therapy Modalities/
4	(physical therap* or physiotherap*).mp.
5	1 or 2 or 3 or 4
6	Arthroplasty, Replacement, Knee/
7	Knee Prosthesis/
8	Knee Joint/
9	Knee/
10	Osteoarthritis, Knee/
11	exp Arthroplasty/
12	Joint Prosthesis/
13	8 or 9 or 10
14	11 or 12
15	13 and 14
16	6 or 7 or 15
17	((arthroplast* or replac* or prosthe*) and knee).mp.
18	16 or 17
19	5 and 18
20	Randomized Controlled Trials as Topic/
21	randomized controlled trial/
22	Random Allocation/
23	Double Blind Method/
24	Single Blind Method/
25	clinical trial/
26	clinical trial, phase i.pt.
27	clinical trial, phase ii.pt.
28	clinical trial, phase iii.pt.
29	clinical trial, phase iv.pt.
30	randomized controlled trial.pt.
31	multicenter study.pt.

© 2019 Buhagiar MA et al. *JAMA Network Open.*

32	clinical trial.pt.
33	exp Clinical Trial as Topic/
34	controlled clinical trial.pt.
35	or/20-34
36	(clinical adj trial$).tw.
37	((singl$ or doubl$ or treb$ or tripl$) adj (blind$3 or mask$3)).tw.
38	PLACEBOS/
39	placebo$.tw.
40	randomly allocated.tw.
41	(allocated adj2 random$).tw.
42	or/36-41
43	35 or 42
44	case report.tw.
45	letter/
46	historical article/
47	or/44-46
48	43 not 47
49	19 and 48

EMBASE

1	exp Rehabilitation/
2	(rehabilit* or habilitat*).mp.
3	exp physiotherapy/
4	(physical therap* or physiotherap*).mp.
5	or/1-4
6	Knee Arthroplasty/
7	Total Knee Replacement/
8	Knee Joint/
9	exp Arthroplasty/
10	Joint Prosthesis/
11	9 or 10
12	8 and 11
13	6 or 7 or 12
14	((arthroplast* or replac* or prosthe*) and knee).mp.
15	13 or 14

© 2019 Buhagiar MA et al. *JAMA Network Open.*

16	5 and 15
17	clinical trial/
18	randomized controlled trial/
19	randomization/
20	single blind procedure/
21	double blind procedure/
22	crossover procedure/
23	placebo/
24	randomi?ed controlled trial$.tw.
25	rct.tw.
26	random allocation.tw.
27	randomly allocated.tw.
28	allocated randomly.tw.
29	(allocated adj2 random).tw.
30	single blind$.tw.
31	double blind$.tw.
32	((treble or triple) adj blind$).tw.
33	placebo$.tw.
34	prospective study/
35	or/17-34
36	case study/
37	case report.tw.
38	abstract report/ or letter/
39	or/36-38
40	35 not 39
41	16 and 40

© 2019 Buhagiar MA et al. *JAMA Network Open.*

eMethods 2. Studies Excluded After Full-Text Review

1. Han A, Nairn L, Harmer A, et al. Early rehabilitation after total knee replacement surgery: a multicenter, noninferiority, randomized clinical trial comparing a home exercise program with usual Outpatient Care. *Arthritis Care Res*. 2015;67(2):196–202.
2. Hensman-Crook A. The effectiveness of physiotherapy intervention with home exercise programme versus patient directed home exercise program following total knee replacement. *Intern Med J.* 2011;41(Suppl. 1):39.
3. López-Liria R, Padilla-Góngora D, Catalan-Matamoros D, et al. Home-based versus hospital-based rehabilitation program after total knee replacement. *Biomed Res Int*. 2015;450421.
4. Mahomed N, Davis A, Hawker G, et al. Inpatient compared with home-based rehabilitation following primary unilateral total hip or knee replacement: a randomized controlled trial. *J Bone Joint Surg Am.* 2008;90:1673-80.
5. Moffet H, Tousignant M, Nadeau S, et al. In-home telerehabilitation compared with face-to-face rehabilitation after total knee arthroplasty: a noninferiority randomized controlled trial. *J Bone Joint Surg Am*. 2015;97:1129-41.
6. Piqueras M, Marco E, Coll M, et al. Effectiveness of an interactive virtual telerehabilitation system in patients after total knee arthroplasty: A Randomized Controlled Trial. *J Rehabil Med.* 2013;45:392–396.
7. Russell T, Buttrum P, Wootton R, Jull G. Low-bandwidth telerehabilitation for patients who have undergone total knee replacement: preliminary results. *J Telemed Telecare.* 2003; 9 (Suppl. 2): S2:44–47

© 2019 Buhagiar MA et al. *JAMA Network Open.*

8. Tousignant M, Moffet H, Boissy P, et al. **A** randomized controlled trial of home telerehabilitation for post-knee arthroplasty. *J Telemed Telecare.* 2011; 17: 195–198
9. Wylde V, Artz S, Dixon E et al. Effectiveness and cost-effectiveness of a group-based outpatient physiotherapy intervention following knee replacement for osteoarthritis: feasibility study for a randomized controlled trial. *Osteoarthritis Cartilage.* 2014;22:S433.

© 2019 Buhagiar MA et al. *JAMA Network Open.*

eTable1. PEDro Scores of Included Studies

Study	Random allocation	Concealed allocation	Groups similar at baseline	Participant blinding	Therapist blinding	Assessor blinding	< 15% dropouts	Intention-to-treat analysis	Between- group difference reported	Point estimate and variability reported	Total (0 to 10)
Buhagiar et al (2017)[16]	Y	Y	Y	N	N	Y	Y	Y	Y	Y	8
Ko et al (2013)[17]	Y	Y	Y	N	N	Y	Y	Y	Y	Y	8
Kramer et al (2003)[32]	Y	N	Y	N	N	Y	N	Y	Y	Y	6
Madsen et al (2013)[33]	Y	Y	Y	N	N	Y	Y	N	Y	Y	7
Mockford et al (2008)[34]	Y	N	Y	N	N	N	Y	Y	Y	N	5
Rajan et al (2004)[35]	Y	N	Y	N	N	Y	Y	Y	Y	Y	7

© 2019 Buhagiar MA et al. *JAMA Network Open.*

eTable 2. Meta-analysis Data

	Studies	Patients	Pooled effect size (95% CI)	P-value	I^2 (%)
Outpatient group or one-to-one physiotherapy compared with home-based program					
Mobility (MD in metres)					
10-12 week follow up	3	373	-11.89 [-35.94, 12.16]	0.33	23
26 week follow up	2	243	-3.05 [-29.75, 23.66]	0.82	0
52 week follow up	3	369	**-25.37 [-47.41, -3.32]**	**0.02**	0
Patient-reported pain and function (SMD)					
10-12 week follow up	4	457	-0.15 [-0.35, 0.05]	0.14	6
26 week follow up	3	313	0.05 [-0.18, 0.28]	0.68	0
52 week follow up	4	515	0.10 [-0.14, 0.34]	0.27	24
Patient-reported quality of life (MD)					
10-12 week follow up	3	314	-0.12 [-0.35, 0.11]	0.31	0
26 week follow up	3	313	-0.16 [-0.48, 0.16]	0.33	45
52 week follow up	2	242	-0.19 [-0.47, 0.08]	0.16	0
Active knee flexion (MD)					
10-12 week follow up	3	386	1.60 [-1.93, 5.13]	0.37	61
26 week follow up	1	114	2.28 [-1.61, 6.16]	0.25	55
52 week follow up	3	386	0.88 [-1.35, 3.10]	0.44	14
Active knee extension (MD)					
52 week follow up	1	143	-0.20 [-1.32, 0.92]	0.73	N/A
Passive knee range of motion (MD)					
10-12 week follow up	3	314	0.30 [-3.85, 4.44]	0.89	0
26 week follow up	3	313	4.22 [-0.33, 8.76]	0.07	0
52 week follow up	2	242	3.40 [-2.52, 9.31]	0.26	0

CI = confidence interval; MD = mean difference; SMD = standardised mean difference

© 2019 Buhagiar MA et al. *JAMA Network Open.*

eFigure 1. Mobility

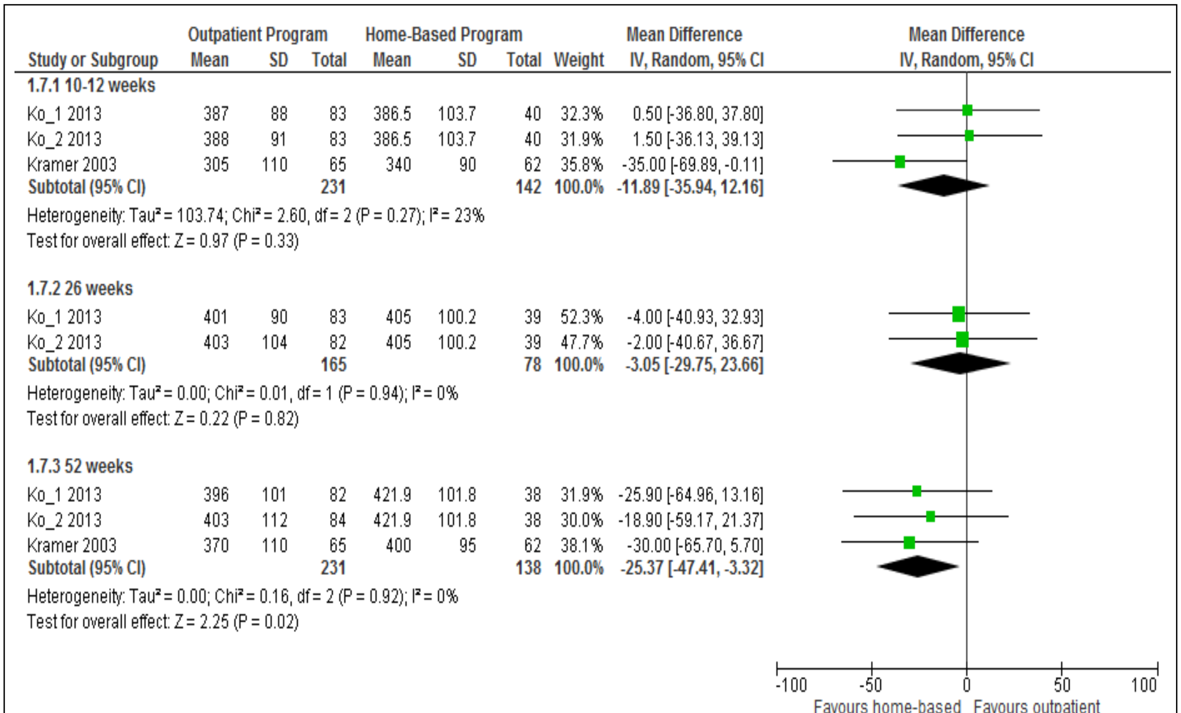

6MWT absolute value: Ko et al 2013, Kramer et al 2003

6MWT = 6 minute walk test; CI = confidence interval; df = degrees of freedom; IV = inverse variance; SD = standard deviation

© 2019 Buhagiar MA et al. *JAMA Network Open.*

eFigure 2. Patient-Reported Pain and Function

Study or Subgroup	Outpatient Program Mean	Outpatient Program SD	Outpatient Program Total	Home-Based Program Mean	Home-Based Program SD	Home-Based Program Total	Weight	Std. Mean Difference IV, Random, 95% CI
**1.1.1 10-12 weeks**								
Ko_1 2013	32.1	7.3	83	33.2	7	40	25.5%	-0.15 [-0.53, 0.23]
Ko_2 2013	33.8	8.1	83	33.2	7	40	25.5%	0.08 [-0.30, 0.45]
Madsen 2013	7.9	7.5	32	8.4	8.8	36	16.4%	-0.06 [-0.54, 0.42]
Mockford 2008	26.3	8.3	71	29.5	9	72	32.6%	-0.37 [-0.70, -0.04]
**Subtotal (95% CI)**			**269**			**188**	**100.0%**	**-0.15 [-0.35, 0.05]**
Heterogeneity: Tau² = 0.00; Chi² = 3.19, df = 3 (P = 0.36); I² = 6%								
Test for overall effect: Z = 1.48 (P = 0.14)								
**1.1.2 26 weeks**								
Ko_1 2013	36	8.4	83	36.4	7.4	39	37.7%	-0.05 [-0.43, 0.33]
Ko_2 2013	37.3	7.7	82	36.4	7.4	39	37.5%	0.12 [-0.26, 0.50]
Madsen 2013	10.5	7.5	34	9.7	8.8	36	24.8%	0.10 [-0.37, 0.57]
**Subtotal (95% CI)**			**199**			**114**	**100.0%**	**0.05 [-0.18, 0.28]**
Heterogeneity: Tau² = 0.00; Chi² = 0.42, df = 2 (P = 0.81); I² = 0%								
Test for overall effect: Z = 0.42 (P = 0.68)								
**1.1.3 52 weeks**								
Ko_1 2013	37.5	7.6	83	38.6	8.2	39	30.8%	-0.14 [-0.52, 0.24]
Ko_2 2013	40.7	6.2	84	38.6	8.2	39	30.7%	0.30 [-0.08, 0.68]
Mockford 2008	25.9	9.3	71	24.7	10	72	38.5%	0.12 [-0.20, 0.45]
**Subtotal (95% CI)**			**238**			**150**	**100.0%**	**0.10 [-0.14, 0.34]**
Heterogeneity: Tau² = 0.01; Chi² = 2.63, df = 2 (P = 0.27); I² = 24%								
Test for overall effect: Z = 0.80 (P = 0.43)								

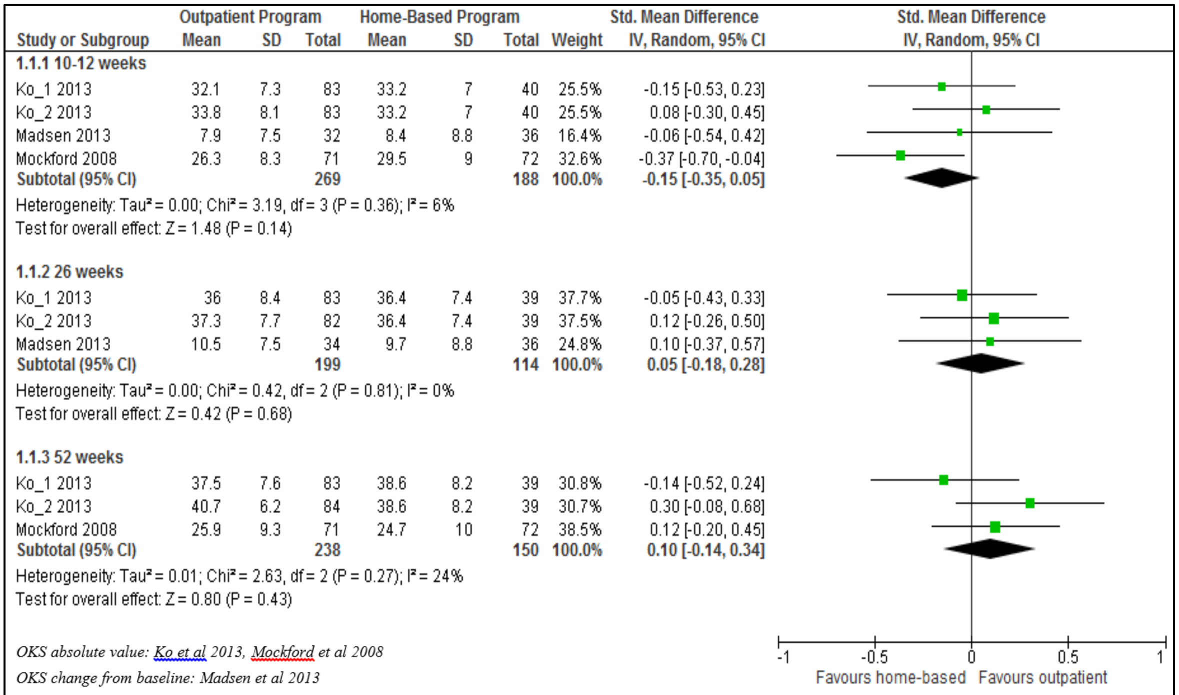

OKS absolute value: Ko et al 2013, Mockford et al 2008

OKS change from baseline: Madsen et al 2013

CI = confidence interval; df = degrees of freedom; IV = inverse variance; OKS = Oxford Knee Score; SD = standard deviation;

© 2019 Buhagiar MA et al. *JAMA Network Open.*

eFigure 3. Patient-Reported Quality of Life

Study or Subgroup	Outpatient Program Mean	Outpatient Program SD	Outpatient Program Total	Home-Based Program Mean	Home-Based Program SD	Home-Based Program Total	Weight	Std. Mean Difference IV, Random, 95% CI
**1.18.1 10-12 weeks**								
Ko_1 2013	9.9	9.9	83	11.3	9.9	40	38.0%	-0.14 [-0.52, 0.24]
Ko_2 2013	9.9	11	83	11.3	9.9	40	38.1%	-0.13 [-0.51, 0.25]
Madsen 2013	16.8	14.2	32	18	16.6	36	23.9%	-0.08 [-0.55, 0.40]
**Subtotal (95% CI)**			**198**			**116**	**100.0%**	**-0.12 [-0.35, 0.11]**
Heterogeneity: Tau² = 0.00; Chi² = 0.05, df = 2 (P = 0.98); I² = 0%								
Test for overall effect: Z = 1.02 (P = 0.31)								
**1.18.2 26 weeks**								
Ko_1 2013	12.5	9.3	83	13.8	9.3	39	36.0%	-0.14 [-0.52, 0.24]
Ko_2 2013	9.9	8.9	82	13.8	9.3	39	35.6%	-0.43 [-0.81, -0.04]
Madsen 2013	22.6	19.4	34	19.5	19.4	36	28.4%	0.16 [-0.31, 0.63]
**Subtotal (95% CI)**			**199**			**114**	**100.0%**	**-0.16 [-0.48, 0.16]**
Heterogeneity: Tau² = 0.04; Chi² = 3.64, df = 2 (P = 0.16); I² = 45%								
Test for overall effect: Z = 0.97 (P = 0.33)								
**1.18.3 52 weeks**								
Ko_1 2013	11.2	10.3	82	13.7	10.7	38	49.7%	-0.24 [-0.62, 0.15]
Ko_2 2013	12	11.5	84	13.7	10.7	38	50.3%	-0.15 [-0.53, 0.23]
**Subtotal (95% CI)**			**166**			**76**	**100.0%**	**-0.19 [-0.47, 0.08]**
Heterogeneity: Tau² = 0.00; Chi² = 0.10, df = 1 (P = 0.75); I² = 0%								
Test for overall effect: Z = 1.40 (P = 0.16)								

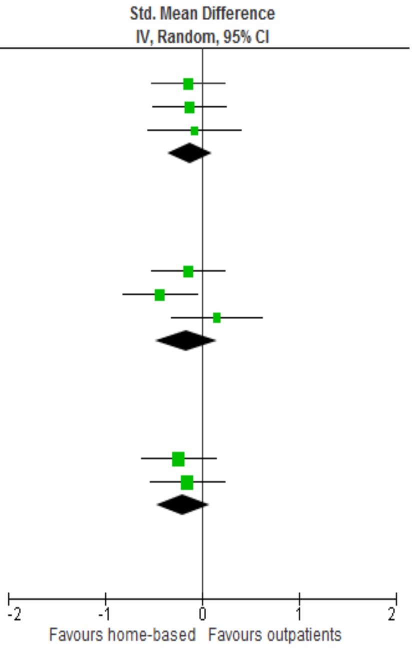

SF12 physical function change from baseline: Ko et al 2013

SF36 physical function change from baseline: Madsen et al 2013

CI = confidence interval; df = degrees of freedom; IV = inverse variance; SD = standard deviation; SF-12 = Short Form 12; SF-36 = Short Form 36;

© 2019 Buhagiar MA et al. *JAMA Network Open.*

eFigure 4. Active Range of Motion

Study or Subgroup	Outpatient Program Mean	SD	Total	Home-Based Program Mean	SD	Total	Weight	Mean Difference IV, Random, 95% CI
**1.13.1 Flexion: 10-12 weeks**								
Kramer 2003	110	14	65	113	14	62	26.8%	-3.00 [-7.87, 1.87]
Mockford 2008	102.1	10.8	71	98.5	11.6	72	34.3%	3.60 [-0.07, 7.27]
Rajan 2004	95	8.5	56	92	8.2	60	38.9%	3.00 [-0.04, 6.04]
**Subtotal (95% CI)**			**192**			**194**	**100.0%**	**1.60 [-1.93, 5.13]**
Heterogeneity: Tau² = 5.93; Chi² = 5.19, df = 2 (P = 0.07); I² = 61%								
Test for overall effect: Z = 0.89 (P = 0.37)								
**1.13.2 Flexion: 26 weeks**								
Kramer 2003	98	10	65	98	14	62	43.1%	0.00 [-4.25, 4.25]
Rajan 2004	97	9	56	93	7.9	60	56.9%	4.00 [0.91, 7.09]
**Subtotal (95% CI)**			**121**			**122**	**100.0%**	**2.28 [-1.61, 6.16]**
Heterogeneity: Tau² = 4.41; Chi² = 2.23, df = 1 (P = 0.14); I² = 55%								
Test for overall effect: Z = 1.15 (P = 0.25)								
**1.13.3 Flexion: 52 weeks**								
Kramer 2003	102	12	65	104	13	62	23.3%	-2.00 [-6.36, 2.36]
Mockford 2008	107.9	11.4	71	106.6	13	72	27.1%	1.30 [-2.71, 5.31]
Rajan 2004	98	8.1	56	96	7.1	60	49.6%	2.00 [-0.78, 4.78]
**Subtotal (95% CI)**			**192**			**194**	**100.0%**	**0.88 [-1.35, 3.10]**
Heterogeneity: Tau² = 0.59; Chi² = 2.34, df = 2 (P = 0.31); I² = 14%								
Test for overall effect: Z = 0.77 (P = 0.44)								
**1.13.6 Extension: 52 weeks**								
Mockford 2008	-1.5	2.2	71	-1.3	2.2	72	100.0%	-0.20 [-0.92, 0.52]
**Subtotal (95% CI)**			**71**			**72**	**100.0%**	**-0.20 [-0.92, 0.52]**
Heterogeneity: Not applicable								
Test for overall effect: Z = 0.54 (P = 0.59)								

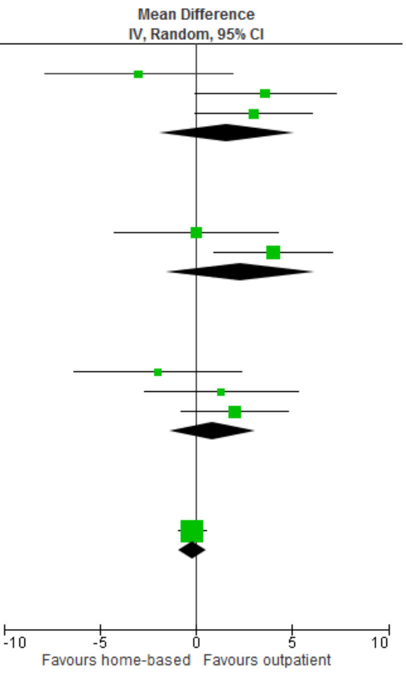

Knee range of motion change from baseline: Kramer et al 2003, Mockford et al 2008, Rajan et al 2004

CI = confidence interval; df = degrees of freedom; IV = inverse variance; SD = standard deviation;

© 2019 Buhagiar MA et al. *JAMA Network Open.*

eFigure 5. Passive Range of Motion Change From Baseline

Study or Subgroup	Outpatient program Mean	Outpatient program SD	Outpatient program Total	Home-Based program Mean	Home-Based program SD	Home-Based program Total	Weight	Mean Difference IV, Random, 95% CI
**1.10.1 10-12 weeks**								
Ko_1 2013	-5.2	27.1	83	-5.2	22.9	40	20.4%	0.00 [-9.18, 9.18]
Ko_2 2013	-4.1	24.9	83	-5.2	22.9	40	21.7%	1.10 [-7.79, 9.99]
Madsen 2013	13.4	12.2	32	13.3	10.5	36	57.9%	0.10 [-5.34, 5.54]
**Subtotal (95% CI)**			**198**			**116**	**100.0%**	**0.30 [-3.85, 4.44]**
Heterogeneity: Tau² = 0.00; Chi² = 0.04, df = 2 (P = 0.98); I² = 0%								
Test for overall effect: Z = 0.14 (P = 0.89)								
**1.10.2 26 weeks**								
Ko_1 2013	5.8	22.3	83	-0.2	24.4	39	25.3%	6.00 [-3.04, 15.04]
Ko_2 2013	5.5	21.1	82	-0.2	24.4	39	26.0%	5.70 [-3.22, 14.62]
Madsen 2013	19.8	14.7	34	17.3	13	36	48.7%	2.50 [-4.02, 9.02]
**Subtotal (95% CI)**			**199**			**114**	**100.0%**	**4.22 [-0.33, 8.76]**
Heterogeneity: Tau² = 0.00; Chi² = 0.52, df = 2 (P = 0.77); I² = 0%								
Test for overall effect: Z = 1.82 (P = 0.07)								
**1.10.3 52 weeks**								
Ko_1 2013	9.5	23.8	82	7.2	21.5	38	47.8%	2.30 [-6.26, 10.86]
Ko_2 2013	11.6	21.1	84	7.2	21.5	38	52.2%	4.40 [-3.79, 12.59]
**Subtotal (95% CI)**			**166**			**76**	**100.0%**	**3.40 [-2.52, 9.31]**
Heterogeneity: Tau² = 0.00; Chi² = 0.12, df = 1 (P = 0.73); I² = 0%								
Test for overall effect: Z = 1.12 (P = 0.26)								

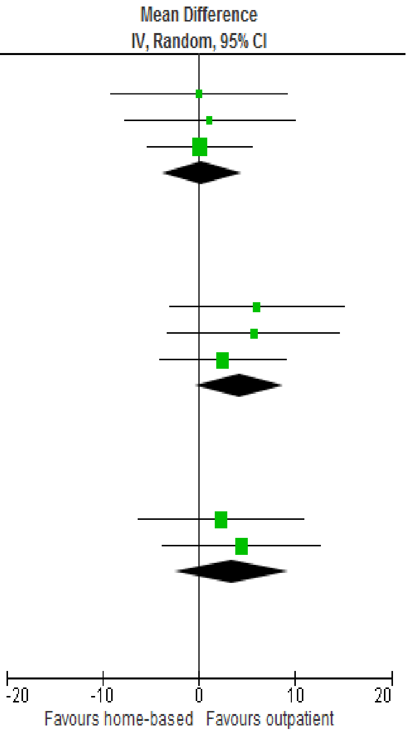

Knee range of motion change from baseline: Ko et al 2013, Madsen et al 2013

CI = confidence interval; df = degrees of freedom; IV = inverse variance; SD = standard deviation

© 2019 Buhagiar MA et al. *JAMA Network Open.*
